# Exploiting the Properties of Non-Wood Feedstocks to Produce Tailorable Lignin-Containing Cellulose Nanofibers

**DOI:** 10.3390/polym16182598

**Published:** 2024-09-14

**Authors:** Meghan E. Lamm, Donna A. Johnson, Katie Copenhaver, Samarthya Bhagia, Amber M. Hubbard, Colleen C. Walker, Kevin Doyle, Soydan Ozcan

**Affiliations:** 1 Manufacturing Science Division, Oak Ridge National Laboratory, 1 Bethel Valley Rd., Oak Ridge, TN 37830, USA; copenhaverke@ornl.gov (K.C.); hubbardam@ornl.gov (A.M.H.); ozcans@ornl.gov (S.O.); 2 Process Development Center, University of Maine, 5737 Jenness Hall, Orono, ME 04469, USA; donna.johnson@maine.edu (D.A.J.); colleen.walker@maine.edu (C.C.W.); kevin.doyle@maine.edu (K.D.); 3 Biosciences Division, Oak Ridge National Laboratory, 1 Bethel Valley Road, Oak Ridge, TN 37831, USA; bhagias@ornl.gov

**Keywords:** lignin-containing cellulose nanofibrils (LCNFs), non-wood feedstock, agricultural residue, hemp, flax, wheat straw, bleached softwood kraft pulp (BSKP)

## Abstract

Lignin-containing cellulose nanofibrils (LCNFs) are mainly produced commercially from treated wood pulp, which can decrease some of the carbon-negative benefits of utilizing biomass feedstock. In this work, LCNFs are prepared from non-wood feedstocks, including agricultural residues such as hemp, wheat straw, and flax. These feedstocks allowed for the preparation of LCNFs with a variety of properties, including tailored hydrophobicity. The feedstocks and their subsequent LCNFs are extensively characterized to determine the roles that feedstocks play on the morphology and properties of their resultant LCNFs. The LCNFs were then incorporated into paper handsheets to study their usefulness in papermaking applications, which indicated good potential for the use of wheat straw LCNFs as a surface additive to improve the oil resistance coating.

## 1. Introduction

Cellulose nanomaterials have revolutionized materials science, due to their numerous potential uses. Specifically, cellulose nanofibrils (CNFs) have served as templates, carriers, and fillers in many applications, and they are also of interest in composite applications, both for reinforcement and as the matrix [1,2,3,4]. For example, reinforcing CNFs have also been used to decrease the coefficient of thermal expansion in various additively manufactured polymer composites, which allows for the production of larger, more complicated structures [5]. Additionally, the use of surface-modified BSKP CNFs as filler in a PLA matrix was able to increase the tensile strength and Young’s modulus by nearly 50% and 500%, respectively, over the neat PLA matrix, and demonstrated over a 20% increase in properties compared to unmodified CNFs [6]. Overall, the performance of CNF reinforcing filler in composites relies on the interfacial forces between the fiber and polymer matrix, which can be affected by the cellulose content of the filler, surface composition, and fiber length (aspect ratio) [7,8]. The presence of lignin can actually improve the interface due to its hydrophobicity, which is similar to many polymer matrices. Lastly, as CNFs generally have longer fibers, and therefore higher aspect ratios, than some reinforcing agents, they tend to work well in composites.

CNFs consist of nanoscale fibrils in the range of 5–50 nm in width and up to several microns in length. To produce CNFs, microfibers are mechanically or chemically fibrillated to separate the agglomerated fibers into individual or branched nanoscale fibers [9]. CNFs can be sourced from a range of materials, including trees, grasses, and agricultural residues [10,11]. However, commercial CNFs are primarily derived from wood sources. Notably, unless the CNF feedstock is extensively processed prior to fibrillation, the resulting products are usually named lignin-containing cellulose nanofibers (LCNFs), due to the contents of lignin and hemicellulose remaining in the sample. LCNFs are unique because the residual lignin can produce special properties, such as hydrophobicity of the resultant CNFs, allowing for more applications.

Researchers have experimented with new feedstocks for CNF and LCNF production throughout the literature, including many waste stream materials. These include sugarcane, kenaf, bamboo, pineapple leaves, oil palm empty fruit bunches, and banana peel [12,13,14,15,16,17,18]. Due to their differences in composition and morphology, CNFs derived from different sources can feature a variety of properties, some of which can be exploited for specific applications. For example, Espinosa et al. produced LCNFs from orange tree pruning and found that their greater delamination helped to reinforce paper more effectively than traditional materials [19]. Prado et al. were able to extract a high yield of pure cellulose nanomaterial from pineapple leaves, as the feedstock itself already contains between 74 and 83 wt.% cellulose [20].

Being able to customize the properties of CNFs using these structure–property relationships between them and their feedstock would be extremely beneficial in optimizing potential usage and applications [21]. Specifically, one use of CNFs is in papermaking, both internally and on the surface of the sheet. They have been shown to increase the tensile index and internal bond when used internally and increase surface properties, including porosity and roughness values, when used on the surface of the sheet [22,23,24]. These property changes can lead to lightweighting of a paper grade and improved coating properties, as well as oxygen barrier and oil and grease barrier properties of various paper grades. In this work, LCNFs were prepared from wheat straw pulp (WS), flax (F), and hemp (H) and compared to control CNFs produced from BSKP, as all three non-wood sources have a fraction of the embodied energy when compared to BSKP (Table 1). Mechanical fibrillation on a disc refining system was used to prepare both the CNFs and LCNFs. Total energy input was used to analyze two samples from each feedstock at a relatively low energy level and a relatively high energy level, to provide two varying degrees of fibrillation. Each of these non-wood LCNFs, and their corresponding CNF controls, were then characterized for composition, morphology, and surface properties, before being incorporated into paper handsheets for further analysis. The structure–property relationships between the feedstocks and their subsequent LCNFs were studied extensively to recommend future applications in papermaking and paper coatings for each customized LCNF material.

## 2. Experimentation

### 2.1. Materials

The following feedstocks were used in this study: Bleached softwood kraft pulp (BSKP), commercially available from Resolute FP (Montreal, QC, Canada), was used to prepare CNF as a control material. Dry lap market pulp; wheat straw pulp; soda cooked pulp; hemp (field-retted European whole hemp plants were washed and chopped to 3 mm pieces); and flax (field-retted European flax was washed and chopped to 3 mm pieces) were all used to prepare LCNFs. All feedstocks were obtained from commercial businesses. Refined hardwood base pulp, commercially available from Woodland Pulp (Baileyville, ME, USA), was used as received.

*Bench-scale fibrillation*: Bench-scale fibrillation was completed using a supermasscolloider (SMC), MKCA6-2 Masuko Sangyo Co., Ltd. (Kawaguchi, Japan) using E6-46 DD plates. The feedstocks were first slushed to defiber the material in an Adirondack pulper, at about 5% solids concentration. Each feedstock was processed through the bench-scale SMC to provide samples at two energy levels: 2500 kW.h/MT and 7000 kW.h/MT net energy, based off dry weight of material.

*Handsheet preparation*: Handsheets were prepared according to TAPPI Standard T205, from blends of 95 wt% refined hardwood base pulp and 5 wt% CNF/LCNF products prepared from various feedstocks. The slurry for the handsheets was prepared by combining the required amount of CNF or LCNF material with the base pulp and mixing in the disintegrator before handsheet production.

### 2.2. Methods

#### Characterization

*Compositional analysis*: The cellulose, hemicellulose, and lignin contents of each dried sample were determined using a two-step sulfuric acid hydrolysis procedure. This procedure involves two acid treatments of lignocellulosic material, followed by high-performance liquid chromatography (HPLC) and UV spectrophotometry, the processes of which are fully described elsewhere [26]. The total lignin content, both acid-soluble and insoluble, as well as cellulose in the form of glucan and hemicellulose as xylan, were quantified.

*X-ray photoelectron spectroscopy (XPS)*: XPS was performed with a Thermo Scientific Model K-Alpha XPS instrument (Waltham, MA USA), which was equipped with microfocused, monochromatic Al Kα X-rays (1486.6 eV) that were focused to a range of spot sizes from 30 to 400 microns. Wide energy range survey spectra (0–1350 eV) were acquired for qualitative and quantitative analysis (pass energy = 200 eV; step size = 1.0 eV). Assessment of chemical bonding of the identified elements was accomplished by collecting core-level spectra over a narrow energy range (pass energy = 50 eV; step size = 0.1 eV). Data were collected and processed using the Thermo Scientific Advantage XPS software package (v. 4.61). When necessary, spectra were charge corrected using the C 1 s core-level peak, set to 284.6 eV. Dilute suspensions of CNFs and LCNFs in water were drop-cast and dried fully for analysis. Feedstocks were analyzed as received.

*Scanning electron microscopy (SEM)*: Dilute suspensions of CNFs and LCNFs in water were drop-cast onto glass slides and dried fully. Dry feedstock was attached to a SEM stage using carbon tape. Both materials were sputtered with iridium and imaged with a Zeiss Merlin VP SEM (Oberkochen, Germany)/scanning transmission electron microscope system equipped with an energy-dispersive X-ray spectroscopy (EDX) unit at an accelerating voltage of 1 keV.

*Energy analysis*: There are two types of energy readings derived from the SMC: net energy and gross energy. Gross energy is the total accumulated amount of energy that the SMC uses, which includes the energy required to run the machine without applying a load to the slurry, termed ‘no load’, and the energy over and above the no load energy imparted to the slurry, termed ‘net energy’, which is applied by ‘loading’ the SMC. Net energy is the difference between gross energy and the amount of energy that is placed on the material when no load is being applied.

Data were collected from the digital power meter in the form of a kilowatt reading at the beginning of each run, with the plates widely separated in the presence of material flow, to determine the “no load” power and obtain the value needed to calculate net energy. After this value was attained, the run began, with load placed on the material by decreasing the gap between the plates at time 0 and every 5 min thereafter; the digital readout on the load meter was documented, then used to calculate the energy during each 5 min interval, as follows:

(1) $$\mathrm{Gross}\;\mathrm{Energy}=\frac{\left(\left(\mathrm{Gross}\;\mathrm{Power}\right)\;\times \;\mathrm{time}\right)\;}{\left(\mathrm{dry}\;\mathrm{weight}\;\mathrm{of}\;\mathrm{material}\right)}=\mathrm{kW}.h/\mathrm{MT}$$

(2) $$\mathrm{Net}\;\mathrm{Energy}=\frac{\left(\left(\mathrm{Gross}\;\mathrm{Power}\;-\;\mathrm{no}\;\mathrm{load}\;\mathrm{Power}\right)\;\times \;\mathrm{time}\right)\;}{\left(\mathrm{dry}\;\mathrm{weight}\;\mathrm{of}\;\mathrm{material}\right)}=\mathrm{kW}.h/\mathrm{MT}$$

*Fines content*: Fines content was defined by the Techpap MorFi Compact (Grenoble, France) fiber analyzer. This machine measures fines as the percentage of total fibril length measured via optical methods that came from fibrils less than 200 µm in length.

*Water retention*: The method used was modified from a method in the literature [27]. Briefly, a 50 mL centrifuge tube, containing 50 g of 1.5% solids content CNF or LCNF suspension in water, was centrifuged at 2000× *g* for 10 min. Water separated from each sample was immediately poured out after centrifugation, and the filtrate (residual CNF or LCNF suspension) was weighed. The water retention of each sample was then calculated according to Equation (3), as follows:

(3) $$\frac{m_{f}}{m_{d}}=\mathrm{water}\;\mathrm{retention}$$

in which *m_f_* refers to the mass of the filtrate and *m_d_* refers to the mass of the dry sample.

*Zeta potential measurements*: Zeta potential measurements were performed in deionized water using a Malvern Zetasizer Pro (Worcestershire, UK) with a minimum of 3 tests to ensure statistical significance. A sample concentration of 0.05% solids was used for each material.

*Solid-state NMR (CP MAS ^13^C SS-NMR) spectroscopy*: Fibrillated biomass CNF/LCNF suspensions in water (flax, hemp, wheat straw pulp, and fibrillated BSKP) were freeze-dried. Non-fibrillated samples (flax, hemp, wheat straw pulp, and BSKP) were in dry form and did not require drying. All these samples were milled in a mini Wiley mill through 40 mesh. These dry and milled samples, except BSKP (no lignin), were delignified using the sodium chlorite/acetic acid (SC/AA) method [28]. Thereafter, 1% biomass was reacted with SC/AA with SC at 0.6 g/g dry biomass of SC and AA at 0.6 mL/g dry biomass of acetic acid at 70 °C for 2 h with intermittent mixing. The delignified samples were washed and dried. These delignified samples, along with BSKP, were then treated with 2.5 M HCl for 4 h at 100 °C to remove hemicellulose, as described previously [28]. These samples, after hemicellulose removal, were washed and freeze-dried, followed by NMR analysis. Solid-state NMR experiments were performed on a Bruker Avance-III 400 MHz (9.4 Tesla) spectrometer (Bruker Biospin Corporation, Bellerica, MA, USA) operating at Larmor frequencies of 100.63 MHz for ^13^C nucleus using a 4 mm double-resonance magic angle spinning (MAS) probe head. The samples were packed into a 4 mm cylindrical zirconia dioxide MAS rotor. The experiments were conducted at room temperature (298 K) at MAS frequencies of 8 kHz. ^1^H-^13^C cross-polarization (CP) with 1.5 ms contact pulse, 3 s recycle delay and 3000 scans were used. The ^13^C chemical shifts were calibrated by externally referencing a methylene signal of adamantane at 38.48 ppm on the tetramethylsilane scale. Non-linear curve fitting with Lorentzian peaks was carried out, and 20-point smoothing was applied using the Savitzky–Golay function. The resulting peak areas of fitted C-4 amorphous and crystalline peaks were used for cellulose crystallinity calculations. The cellulose crystallinity index (CrI) was determined from the integration areas of the crystalline and amorphous C-4 signals of CP/MAS ^13^C NMR spectra using Equation (4), as follows:

(4) $$CrI=\frac{A_{86–92\mathrm{ppm}}}{A_{79–86\mathrm{ppm}}+A_{86–92\mathrm{ppm}}}\times 100$$

where A_86–92ppm_ and A_79–86ppm_ are the areas of the crystalline and amorphous C-4 carbon signals of cellulose, respectively.

*Thermogravimetric analysis (TGA)*: The degradation temperatures were determined by TGA using a TA Instruments Q500 apparatus (New Castle, DE, USA); the degradation temperature (T_d_) was defined as the temperature at which a 5 wt.% mass loss was experienced by the sample. The samples were heated from room temperature to 100 °C at a ramp rate of 10 °C/min and held at isotherm for 10 min to remove water, and the sample was then heated to 600 °C at a ramp rate of 10 °C/min, all of which occurred under nitrogen flow. Dilute suspensions of LCNFs in water were drop-cast and dried fully for analysis. Feedstocks were analyzed as received.

*Freeness*: The desired amount of LCNF material was combined with a refined hardwood base pulp and mixed in a standard handsheet disintegrator. This slurry was measured for Canadian Standard Freeness according to TAPPI Standard T227. Freeness was measured on a Canadian Standard Freeness Tester made by Star Brass Manufacturing Co. (Boston, MA, USA); 2 replicates, within 2% error, were performed as directed by the test standard.

*Tensile index*: Performed according to TAPPI Standard T494. An Instron tensile tester by the Instron company (Norwood, MA, USA) was used to measure tensile index; 10 replicates, within 8% error, were performed as directed by the test standard.

*Internal bond*: Performed according to TAPPI Standard T569. Internal bond by Testing Machines, Inc. (New Castle, DE, USA) was used to measure the internal bond; 10 replicates, within 8% error, were performed as directed by the test standard.

*Porosity*: Performed according to TAPPI Standard T47. Porosity was measured on a genuine Gurley 4340 Automatic Densometer by Gurley Precision Instruments (Troy, NY, USA); 10 replicates, within 8% error, were performed as directed by the test standard.

*Roughness*: Performed according to TAPPI Standard T538. A smoothness tester “Precisionaire” by Sheffield Corporation, Precision Gage & Tool Co. (West Carrollton, OH, USA) was used to measure the roughness; 10 replicates, within 8% error, were performed as directed by the test standard.

*Contact angle measurements*: Contact angle measurements were performed on a Kruss 25E Drop Shape Analyzer (Hamburg, Germany) using water as the medium. Angles were taken after 5 s of equilibration and determined using an ellipsoid shape fitting. At least 5 replicates were analyzed. Samples were prepared by drop-casting a dilute suspension of CNF/LCNF material in water on glass slides and drying fully on a hot plate.

## 3. Results

Three different natural fibers (wheat straw, hemp, and flax) were chosen for this study, due to their availability, and a comparison was made to traditional bleached softwood kraft pulp. These feedstocks were chosen due to a rising interest in utilizing sustainable materials and agricultural byproducts, such as flax and wheat straw, and locally available options, such as hemp.

### 3.1. Feedstock Analysis

**Figure 1:**
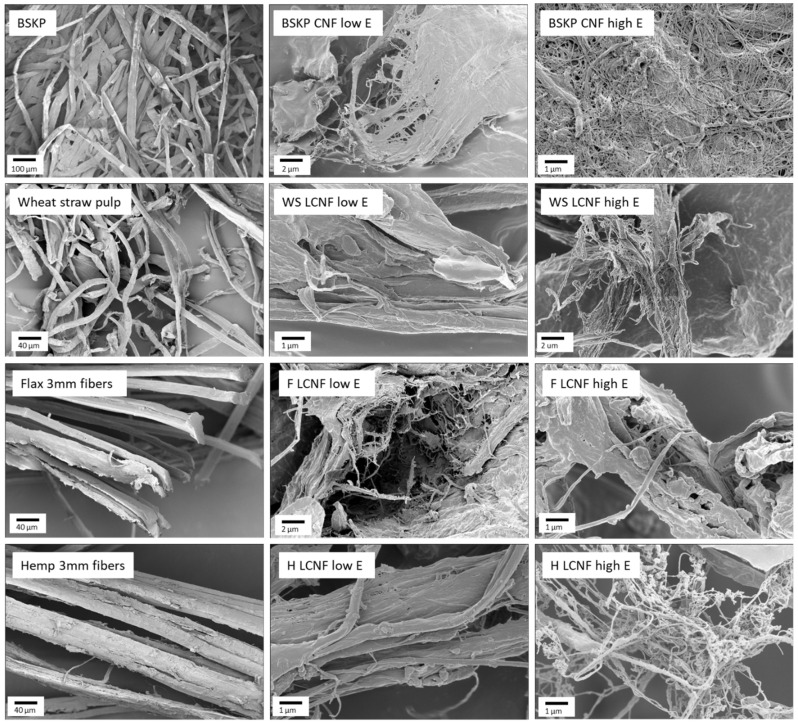
Scanning electron microscopy images of the non-wood feedstocks and their subsequent LCNFs at low and high net processing energy. WS LCNFs, F LCNFs, and H LCNFs refer to LCNFs derived from wheat straw, flax, and hemp, respectively.

In order to understand the structure–property relationships between the feedstocks and their resultant CNF or LCNF products, extensive initial characterization was performed on the feedstocks. Firstly, the compositions of the feedstocks were analyzed using a variety of methods (Table 2). Due to the pulping process and subsequent bleaching, the BSKP contained a large amount of cellulose and no lignin. Comparatively, both hemp and flax contained similar amounts of cellulose, but also contained significant portions of lignin and minimal hemicellulose. Wheat straw pulp contained the least amount of cellulose and equal amounts of hemicellulose and lignin. Flax and hemp also contained higher amounts of extractives, consistent with their decreased level of processing. These results are all expected based on the literature and considering the structures of the feedstocks versus the roles of cellulose, hemicellulose, and lignin within them [29,30,31]. These compositional data were also consistent with the XPS results (Table S1), which indicated that BSKP contained larger amounts of cellulose, as shown by large C1s C-OH and O1s O-C peaks, when compared to hemp and flax. Likewise, hemp and flax had much larger C1s C-C peaks, consistent with the greater fractions of lignin in their composition. Wheat straw and WS LCNFs also had clear peaks for the presence of silica, which did not appear in the other feedstocks or their resultant LCNFs. All feedstocks and their LCNFs contained calcium, while only hemp had a large phosphorus peak (Figure S3). It is worth noting that the cellulose contents for wheat straw, flax, and hemp differed between measurement techniques. Differences between the compositional data and XPS results could be because XPS is only a surface-level measurement, potentially limiting its effectiveness. Scanning electron microscopy (SEM) images were also collected for the feedstocks (Figure 1). All feedstocks displayed longer fibers (i.e., widths greater than a few microns), rough surfaces, and branching morphology. However, it is worth noting that the BSKP appears the most fibrillated on SEM. It is noteworthy that a full pulping process was completed on both the BSKP and wheat straw prior to processing, while the hemp and flax were only field retted, allowing enzymes to break down some of the initial chemical bonds present. To overcome this difference, all feedstocks were slushed to defiber any agglomerates. The authors feel it is important to compare processed BSKP to underprocessed non-wood feedstocks, as this saves significant embodied energy in material processing, improving the carbon footprints of these feedstocks.

### 3.2. CNF/LCNF Production and Analysis

**Figure 2:**
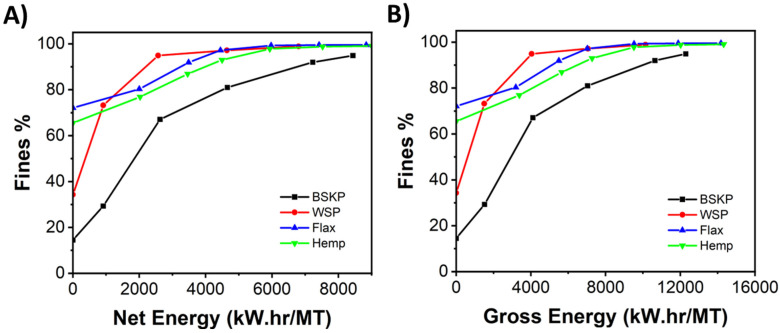
(**A**) Net energy versus fines content and (**B**) gross energy versus fines content.

CNF/LCNF materials were produced via mechanical fibrillation with a bench-scale supermasscolloider (SMC), at 1.5 wt.% for the BSKP and 3 wt% for the non-wood feedstocks, in water. Due to the presence of lignin and hemicellulose, the viscosity levels of the non-wood feedstocks were much lower than that of BSKP, allowing them to be processed at a much higher solids contents to achieve similar viscosity. Fines % is used as a process control measure during the production of CNF/LCNF materials, since it is a straightforward, fast, and replicable measurement that can be performed during the production process. The fines % is the total percentage of sample which contains fibers under 0.2 mm in length [32]. Figure 2 shows the fines vs. energy curve generated in the production process, considering both net energy and gross energy. Both show increasing fines content with increasing energy input. The net energy, the energy that goes into the pulp, relates more to the properties of the pulp (while the gross energy is the total energy used in the process). The net energy curves of the different feedstocks show that the more processed feedstocks, bleached softwood kraft pulp and wheat straw pulp, have lower levels of fines at the outset of the run, while the hemp and flax have higher fines contents before refining. Refining of all feedstocks results in increasing fines content, with the non-wood feedstocks’ fines content leveling off at near 100% fines at a lower energy level than the BSKP. While the fines measurement is a good process control measurement, it is not a good indicator of the nanomorphology of the CNF/LCNF materials, since it is an optical measurement, measuring down to 2 microns in size. This measurement also does not necessarily correlate well with product performance.

**Figure 3:**
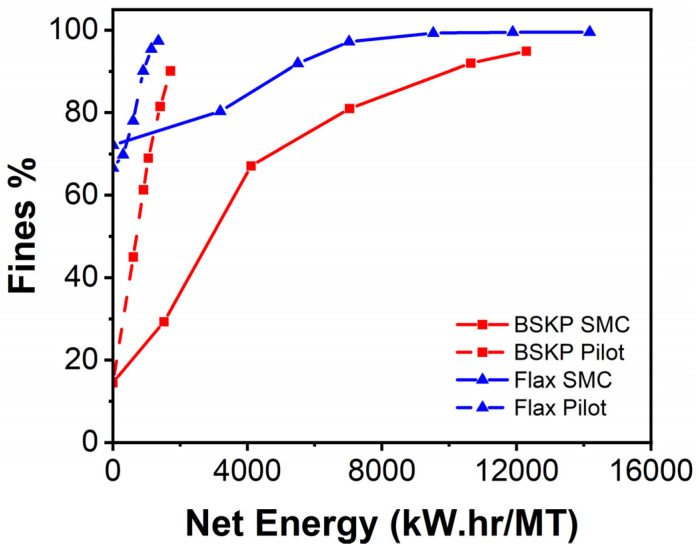
Net energy for bench SMC and pilot scale 20SD CNF/LCNF production versus fines content.

This study used the bench-scale SMC for comparative work evaluating non-wood feedstocks versus BSKP. While the energy data collected were comparative between feedstocks for this study, they do not represent the energy requirements to produce CNF/LCNF materials on a commercial scale. Other work has been done on the flax feedstock on a commercial scale 20″ disk refiner (Figure 3). This shows that the commercial scale refining energy is much lower than the bench-scale energy, but the relative energies between feedstocks seem similar at both scales.

The CNF/LCNF materials were characterized for a number of properties. For viscosity, water retention, freeness, and handsheet work, data were collected from CNF/LCNF samples at several production energy levels. For other tests, such as SEM, XPS, etc., CNF/LCNF samples produced at two net energy levels were chosen, 2500 kW.h/MT (low) and 7000 kW.h/MT (high). The resulting CNF/LCNF materials formed gels in water at 3 wt.% (Figure S1). This likely indicates some fibrillation has occurred, but additionally that the LCNFs swelled in contact with water, causing a gel phase between the nanoscale fibers and surrounding water.

SEM images were taken of each sample after fibrillation with low and high energy, where all CNF/LCNF samples displayed clear fibrillar morphology that features nanoscale dimensions in contrast to the feedstock materials. The SEM images indicated that the non-wood feedstocks required more energy to reach this fibrillar state, with defined nanoscale dimension only being observed in the high-energy samples, whereas BSKP samples were nanoscale fibrils at both energy levels. It is also worth noting that the fibrillation processes in the wheat straw, hemp, and flax displayed the same pattern. At low fibrillation energy, the main fibers appeared to be broken into smaller branching fibers, while, at high fibrillation energy, these bundles of branching fibers appeared to be separated into free-standing nanoscale fibers. XPS was used to study the composition, the results of which are listed in Table S2. Compared with their respective feedstocks, the BSKP CNFs, flax LCNFs, and hemp LCNFs have much higher levels of C1s C-O and O1s O-C, which is consistent with the presence of more hydroxyl groups at the surface. These values for hemp LCNFs increased with a higher net energy, consistent with an increasing surface exposure (i.e., degree of fibrillation). Flax differed, as the C1s C-O and O1s O-C prevalence remained consistent regardless of refining. The flax also appears to have fibrillated the least according to Figure 1, so this lack of change is unsurprising. As for wheat straw, there were decreases in the C1s C-O and O1s O-C, so we suspect that the pulping process produces a high number of surface hydroxyl groups of cellulose, lignin, and hemicellulose. During fibrillation, however, the cellulose fibrillation produces additional surface hydroxyl groups, but likely also changes the surface chemistry on the LCNFs, causing the initial decreases in C1s C-O and O1s O-C, and then subsequent increases with increasing energy levels.

**Figure 4:**
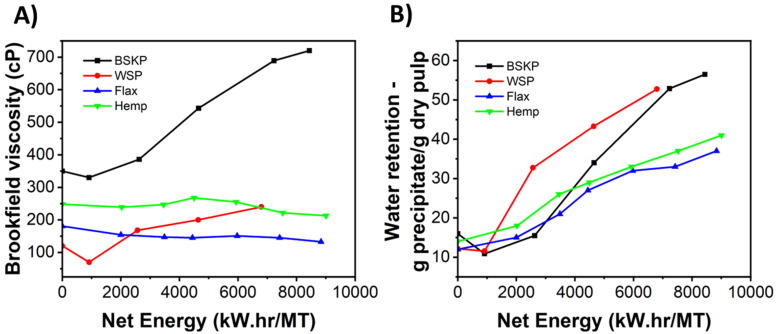
(**A**) Net energy versus viscosity, measured using a Brookfield viscometer, and (**B**) net energy versus water retention for BSKP and the non-wood feedstocks during CNF/LCNF processing.

Next, the viscosity of each CNF/LCNF solution after fibrillation was taken, as it can indicate the degree of fibrillation within a sample (Figure 4A). The BSKP displayed the highest overall viscosity at each energy level tested, which seems incongruous with the fines measurement. Since fines content is not a great estimation of fibrillation, it is more important to analyze the effect of structure on viscosity. It is likely that the presence of hydrogen bonding between cellulose hydroxyl groups and water can increase a sample’s viscosity. These interactions were likely more present in the BSKP samples than the non-woods due to a lack of lignin to disrupt bonding. However, the wheat straw samples do increase in viscosity with increasing net energy. This is likely attributable to increased fibrillation, as the presence of lignin would disrupt some of the hydrogen bonding seen in BSKP. Lastly, flax and hemp appear to remain plateaued in viscosity across all energy levels, after initially being higher than in wheat straw. This is consistent with their fines contents and is likely due to the initial fibrous morphology of their feedstocks, which remained in both samples at either energy level on SEM. Water retention in each of the CNF/LCNF materials was measured, indicating how strongly the CNF/LCNF structures hold on to water during centrifugation, due in part to fiber swelling and fibrillation (Figure 4B). Here, water retention for the wheat straw pulp and the bleached softwood pulp increases more at higher energy levels than for the hemp and flax. The results indicate a higher level of fibrillation in BSKP and wheat straw pulp compared to in hemp and flax. It should be noted that this difference in water retention can be visually seen in Figure S1, as well. Finally, the zeta potential values for the traditional BSKP-sourced CNFs are noticeably lower compared to those of the LCNFs sourced from non-wood feedstocks (cf. Figure S2), further validating these results. The low value for BSKP indicates that it is stable in water, likely due to the increased fibrillation on the surface that produces more hydroxyl groups and causes this stabilization. Likewise, the poorer stability of WS LCNFs is likely due to the high presence of lignin, which is unstable in water due to its hydrophobicity. Flax and hemp LCNFs lie in the middle, due to the presence of lignin, but still very high levels of LCNFs and, therefore, surface hydroxyl groups, due to fibrillation.

Lastly, the crystallinity and thermal degradation behaviors of all CNF/LCNF materials were compared (Table 3). Crystallinity was determined using ss-NMR, shown in Figure S4. Cellulose in flax and hemp, both non-fibrillated and fibrillated, had higher crystallinity than that in wheat straw and BSKP. This could be inherent to the material, or could be caused by the lack of pulping of both feedstocks prior to fibrillation. Crystallinity changes with fibrillation did not show a common trend among the four kinds of cellulose sources, but were consistent with values found in the literature [33,34]. There appear to be competing mechanisms, like shearing forces and drying-induced hornification, that have varying effects depending on the nature of linkages of cellulose with hemicellulose and lignin [35]. Thermal stability of the CNF/LCNF materials decreased with increasing fibrillation across all feedstocks, probably because of increases in surface area due to fibrillation that reduced mass transfer resistance to degradation. BSKP and its CNFs had the highest stability values, which is unsurprising, as it did not contain hemicellulose, the least thermally stable constituent of lignocellulosic biomasses [36]. However, the lignin content and its phenolic structure accounts for a large amount of the char yield, which is why the char yield was lowest for the BSKP samples. This also explains the high char yields in wheat straw, hemp, and flax, all of which have significant amounts of lignin by composition. The pattern in lignin content between these materials is also consistent with the patterns in their char yields. Meanwhile, their contents of hemicellulose explain their lower thermal stability compared to BSKP.

### 3.3. CNF/LCNF Materials in Paper

To help determine the degree of fibrillation and show a usage case for CNF/LCNF materials, handsheets of paper were prepared. In order to assess the effects of CNF/LCNF materials on the properties of paper, handsheets were prepared using 95% refined hardwood base pulp, mixed with 5% LCNFs, then select strength, porosity, and surface properties were measured. A variety of paper-making characterizations were completed to study the effects of feedstock and fibrillation level.

**Figure 5:**
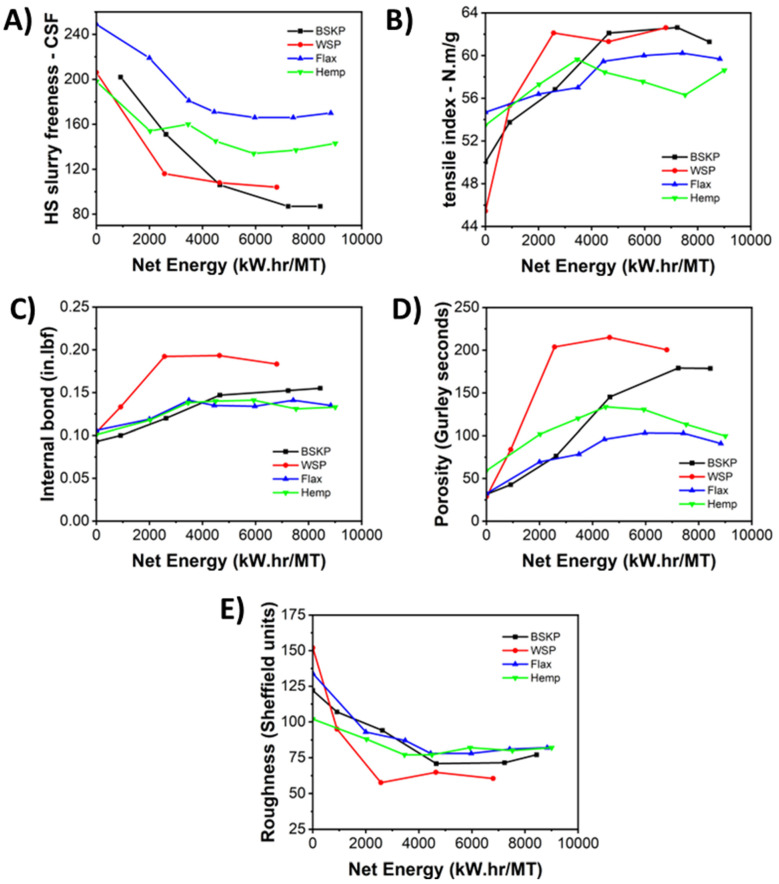
Characterization of handsheets containing 5 wt.% CNFs or LCNFs, collected at varying energy level including (**A**) freeness, (**B**) tensile index, (**C**) internal bond, (**D**) porosity, and (**E**) roughness.

Firstly, Canadian Standard Freeness was measured on the blended hardwood/CNF/LCNF handsheet slurry. The blended freeness, while not a direct measurement on the CNF/LCNF materials themselves, gives an indication of their effects on the handsheet slurry and the subsequent handsheet. Freeness measures how easily the water drains from the pulp suspension, containing pulp and CNF/LCNF materials (Figure 5A). This relates to the swelling and fibrillation of the fibers—as the level of papermaking stock prep refining and level of fibrillation increase, the freeness will decrease. The freeness also relates to the properties of the pulp—increasing refining, hence decreased freeness, will increase paper strengths. In this study, the decreasing blended freeness would indicate that the BSKP CNFs and WS LCNFs are both fibrillated more than those of the hemp and flax, consistent with the water retention data discussed earlier.

Next, the strength of each handsheet was determined using tensile index and internal bond (Figure 5B,C). In handsheets, strength is important because paper undergoes structural failure due to stresses, impacts, and loading, both in and out of plane [37]. During the papermaking stock preparation process, the fiber is refined to increase fibrillation and fiber surface area, which promotes hydrogen bonding, and improves paper properties. Internal properties of the paper sheet include tensile and internal bonds, which are improved with increased refining. Surface properties of the paper sheet, such as porosity and surface roughness values, are also improved with refining. Adding CNF/LCNF materials to the handsheet showed increasing tensile index, internal bond, and porosity values, as well as decreasing roughness values, with increasing LCNF processing energy.

The tensile index developed higher values for the BSKP and WS handsheets than either the hemp or flax handsheets as the levels of energy and processing of CNF/LCNF materials added to the handsheets increased, consistent with previous data. For hemp and flax, the lower tensile indices at higher energy levels could be due to poor adhesion between the LCNFs and hardwood pulp, potentially due to their increased levels of lignin and hemicellulose, and the decreased level of fibrillation compared to the other materials. Internal bond values were higher for the WS, with the other three LCNF feedstock handsheets showing generally similar values, although the BSKP showed increasing values with increasing CNF processing energy, indicative of increased fibrillation. Internal bond looks specifically at bonding between the fibers in the Z direction. More fibrillated fibers tend to have better interactions, but the composition of the fibers also plays a role in their interactions. WS displayed the highest internal bond, consistent with its higher level of fibrillation, but the presence of lignin and hemicellulose likely strengthens the interface, which is why the other three feedstocks, containing less lignin and hemicelluloses, display similar properties.

Lastly, the porosity and roughness of the handsheets were determined (Figure 5D,E). Porosity measures the amount of time it takes for a given amount of air to move through the sheet, so higher values indicate a ‘tighter’, less porous sheet, wherein it takes more time for air to flow through the sheet. CNF/LCNF materials are important for this property because their great surface area creates a better bonding network, preventing movement of the air through the sheet. Additionally, the presence of hydrophobic moieties, like lignin, act as sizing agents and can prevent the movement of air, as air contains water vapor and tends to be hydrophilic. WS performed the best with this measurement, due to its high degrees of fibrillation and composition. However, the high degree of fibrillation in higher energy level BSKP also performed quite well, and outperformed both hemp and flax. Roughness measurements examine the surface of the paper, and lower roughness values indicate a smoother sheet. A paper surface has topography to it, made by the intersections of the fibers making up the sheet. This can be controlled by the amount of bonding between fibers, and by materials filling in the holes between fibers. If the holes are filled in, then coating formulations applied to smoother paper surfaces could be more effective as gas barriers [38]. WS LCNF handsheets displayed a significantly lower roughness (higher smoothness), while the rest of the feedstocks were all similar and, overall, featured low roughness, likely due to the presence more microscale fibers capable of filling holes on the surface of the sheet.

It was assumed, from the data collected, that all three non-wood LCNFs would be hydrophobic in nature, due to their lignin contents. To this extent, the higher content of lignin in the WS LCNFs likely accounts for some of its improved performance versus other samples. To confirm this hypothesis, water contact angles were collected for the samples (Figure S5). Unsurprisingly, both flax and hemp LCNFs displayed hydrophobic results, with a water contact angle above 100° for both samples, at 113 (±7) and 108 (±2)°, respectively, consistent with their levels of lignin and extractives. Additionally, these results are consistent with LCNFs produced from hemp in the literature [39]. However, both BSKP and WS LCNFs were hydrophilic according to this measurement, at 68 (±3) and 64 (±4)°, respectively. As WS LCNFs have the highest content of lignin among all four samples, this was shocking. While it is uncertain why this result occurred, it is believed to be related to the soda cook which occurred on the WS that results in condensation and relocation of the lignin, likely producing these unique results.

It was, therefore, assumed that WS LCNFs could behave as an oil barrier on paper, due to their increased hydrophilicity at the surface compared to even BSKP CNFs. LCNFs have been shown in the literature to serve as a good grease barrier [40]. Additionally, in the literature, LCNFs have been shown to serve as a good water and air vapor barrier, especially when compared to BSKP CNFs [41,42]. Our results for porosity indicate a potential for good air vapor barrier properties, consistent with that research, but additional testing outside of the scope of this work would be required. Likewise, as CNFs have shown promise in many composite applications, the differences in hydrophobicity of the various CNFs and LCNFs produced in this study could prove useful as reinforcement in a variety of polymer matrices, which, themselves, tend to be hydrophobic. For example, one study found that, as reinforcement in polymer composites, LCNFs outperformed CNFs in regards to tensile properties, with and without hemicellulose, in a PLA matrix [43]. This is, however, out of scope for this study and could be included in further research.

## 4. Conclusions

LCNFs were produced from three non-wood feedstocks and compared to CNFs produced from standard BSKP. These CNFs and LCNFs were extensively characterized for their composition, morphology, and thermal degradation. Handsheets were then prepared, using the CNFs and LCNFs for comparison, in paper applications, which were characterized to help determine fibrillation levels and usefulness in this application. From all the characterizations, it is seen that BSKP and WS displayed higher levels of fibrillation than hemp and flax, with WS LCNFs achieving their nanoscale morphology at a lower net energy level, indicating faster fibrillation time. Additionally, the WS LCNFs displayed good wet additive properties, as it appears the WS LCNFs can aggregate along the surface of handsheets with good gas and oil resistance, suggesting their potential use in coating applications. In future studies, initial pulping of the hemp and flax, prior to fibrillation, will be attempted to elucidate the effect of pulping on fibrillation as well, since that could have played a role in the positive performance of WS. Overall, this study indicates that non-wood sources can be used to produce LCNFs with tailorable properties, helping to push this valuable resource towards new applications or improvement in current applications.

## Figures and Tables

**Table 1:** Embodied energy of the various feedstocks used in this work [25].

Feedstock	Embodied Energy (MJ/kg)
BSKP	37.3
Wheat straw	18.8
Flax	10–12
Hemp	9.5–10.5

**Table 2:** Compositions of the feedstocks used in this study to produce LCNFs.

Material	Cellulose %	Hemicellulose %	Lignin %	Extractives %
Bleached softwood kraft pulp (BSKP)	79.3 (±1.7)	18.4 (±0.7)	0	2.3 (±1.2)
Wheat straw pulp (WS)	49.9 (±0.9)	21.8 (±0.5)	24.8 (±2.2)	3.5 (±1.2)
Flax (F)	73.5 (±0.7)	7.0 (±1.7)	15.3 (±0.3)	5.9 (±0.8)
Hemp (H)	77.4 (±3.8)	5.4 (±0.8)	11.2 (±0.3)	6.1 (±1.6)

**Table 3:** Cellulose crystallinity and thermal decomposition behaviors of non-wood feedstocks and CNF/LCNF materials, measured using solid-state nuclear magnetic resonance spectroscopy (ssNMR) and thermal gravimetric analysis (TGA).

Material	Cellulose Crystallinity %	T_d_ (5%, °C)	Char Yield (%)
BSKP	42.9	304	12.3
BSKP CNFs low E	45.0	293	15.9
BSKP CNFs high E	41.8	292	16.5
WS pulp	40.0	271	20.5
WS LCNFs low E	44.3	236	30.8
WS LCNFs high E	47.9	230	32.6
Flax	70.0	273	18.8
Flax LCNFs low E	73.8	242	25.8
Flax LCNFs high E	68.2	269	27.4
Hemp	67.9	278	20.1
Hemp LCNFs low E	61.8	265	24.2
Hemp LCNFs high E	63.0	250	28.0

## Data Availability

The original contributions presented in the study are included in the article/Supplementary Material, further inquiries can be directed to the corresponding author.

## Supplementary Materials

The following supporting information can be downloaded at: https://www.mdpi.com/article/10.3390/polym16182598/s1, Figure S1: Photographs of the CNF/LCNF and feedstocks used to produce them. The pictures on the right side are CNF/LCNF suspensions in water containing about 3 wt.% solids; Figure S2: Zeta potential of the LCNF produced from various feedstocks. Values less than -30 mV indicate greater stability in the medium, water; Figure S3: Entire XPS spectra of the various CNF and LCNF produced in this study. Note, only BKSP low energy is shown as both the low and high energy samples appeared the same on XPS; Figure S4: ^13^C ss-NMR CP/MAS spectra of cellulose extracted from various biomass samples; Figure S5: Water contact angle for the various CNF and LCNF produced in this work; Table S1: XPS peaks from the C 1s and O 1s regions for the various feedstocks; Table S2: XPS peaks from the C 1s and O 1s regions for the various CNF and LCNF.
