# Supplementary material for: Exploiting the Properties of Non-Wood Feedstocks to Produce Tailorable Lignin-Containing Cellulose Nanofibers
Source: Polymers (Basel). 2024 Sep 14;16(18):2598. doi: 10.3390/polym16182598 (PMC11435538; doi:10.3390/polym16182598)
Supplement: Supplementary file 1 [file polymers-16-02598-s001.zip › polymers-3152411-supplementary.pdf]

Exploiting the Properties of Non-Wood Feedstocks to Produce Tailorable Lignin-containing Cellulose Nanofibers

Meghan E. Lamm [1,*], Donna A. Johnson [2], Katie Copenhaver [1], Samarthya Bhagia [3], Amber M. Hubbard [1], Colleen C. Walker [2], Kevin Doyle [2] and Soydan Ozcan [1]

[1] Manufacturing Science Division, Oak Ridge National Laboratory, 1 Bethel Valley Rd. Oak Ridge, TN 37830, USA; copenhaverke@ornl.gov (K.C.); hubbardam@ornl.gov (A.M.H.); ozcans@ornl.gov (S.O.)
[2] Process Development Center, University of Maine, 5737 Jenness Hall, Orono, ME 04469, USA; donna.johnson@maine.edu (D.A.J.); colleen.walker@maine.edu (C.C.W.); kevin.doyle@maine.edu (K.D.)
[3] Biosciences Division, Oak Ridge National Laboratory, 1 Bethel Valley Road, Oak Ridge, TN 37831, USA; bhagias@ornl.gov
* Correspondence: lammme@ornl.gov

Supporting Information

Table S1. XPS peaks from the C 1s and O 1s regions for the various feedstocks.

Material	Peak	Binding energy (eV)	Atomic %
**Bleached softwood Kraft pulp (BSKP)**	C1s C-C	284.6	5.0
	C1s C-O	286.5	27.0
	C1s C=O	287.8	21.3
	C1s carboxyl	289.3	4.7
	O1s O-C	533.0	27.8
	O1s O=C	534.1	14.1
**Wheat straw pulp**	C1s C-C	284.3	21.3
	C1s C-O	286.0	32.1
	C1s O-C-O	287.4	10.3
	C1s carboxyl	288.8	1.7
	O1s O-C	532.4	33.1
	O1s O=C	-	-
**Flax**	C1s C-C	284.3	55.7
	C1s C-O	285.9	17.6
	C1s O-C-O	287.3	6.1
	C1s carboxyl	288.5	2.4
	O1s O-C	532.3	13.5
	O1s O=C	531.4	2.5
**Hemp**	C1s C-C	284.5	53.6
	C1s C-O	285.6	15.9
	C1s O-C-O	287.0	7.1
	C1s carboxyl	288.6	4.5
	O1s O-C	532.9	8.0
	O1s O=C	531.8	8.1

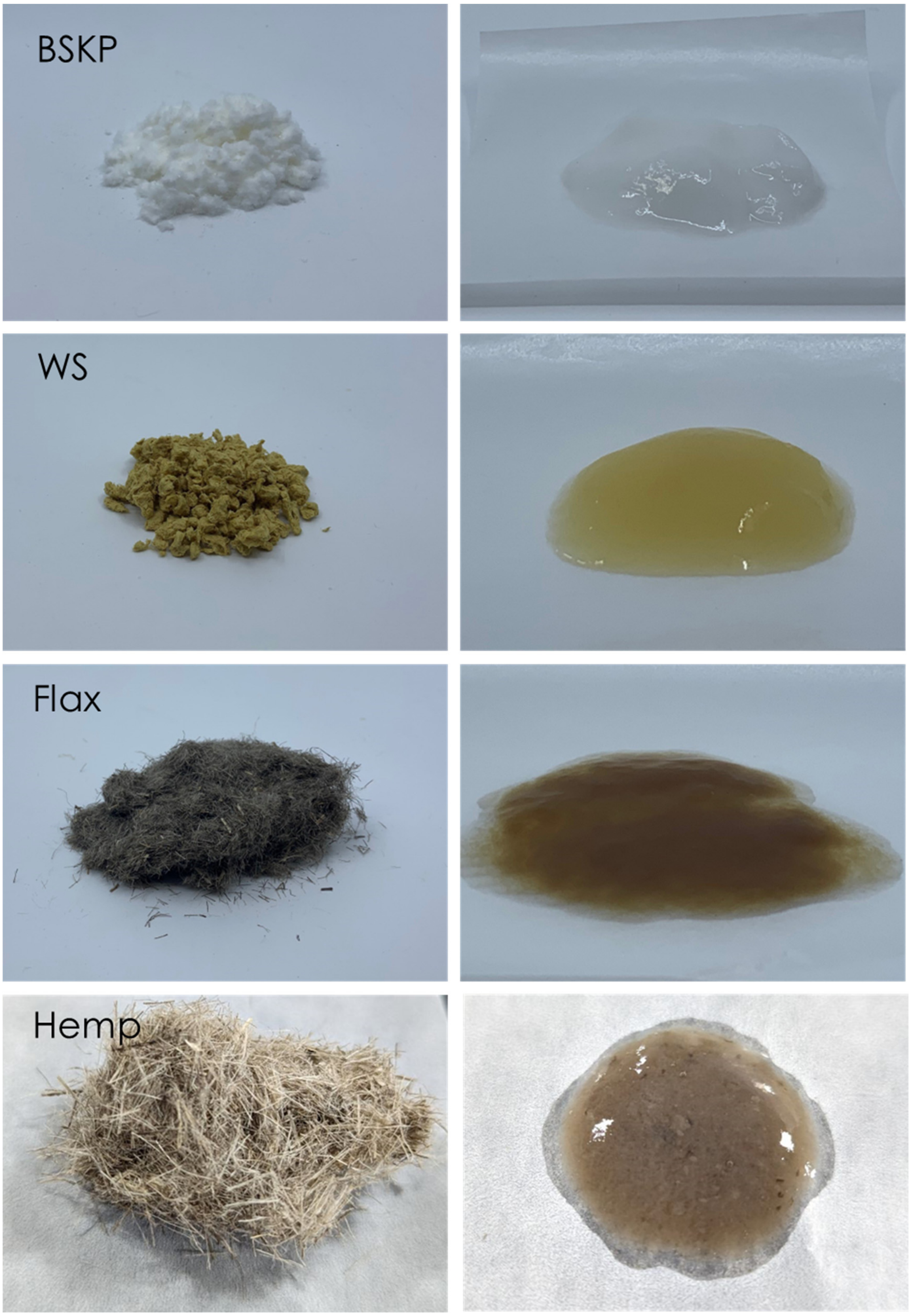

Figure S1. Photographs of the CNF/LCNF and feedstocks used to produce them. The pictures on the right side are CNF/LCNF suspensions in water containing about 3 wt.% solids.

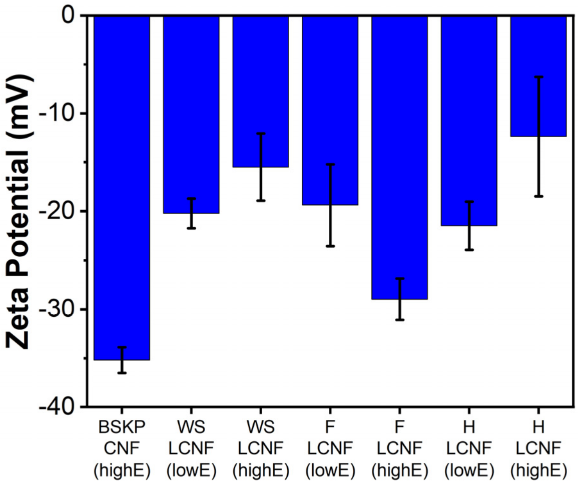

Figure S2. Zeta potential of the LCNF produced from various feedstocks. Values less than -30 mV indicate greater stability in the medium, water.

Table S2. XPS peaks from the C 1s and O 1s regions for the various CNF and LCNF.

Material	Peak	Binding energy (eV)	Atomic %
**BKSP CNF**	C1s C-C	284.6	14.8
	C1s C-O	286.3	35.8
	C1s O-C-O	287.9	9.3
	C1s carboxyl	289.1	1.2
	O1s O-C	532.7	38.7
	O1s O=C	-	-
**WS LCNF Low E**	C1s C-C	284.6	38.5
	C1s C-O	286.2	24.2
	C1s O-C-O	287.7	7.1
	C1s carboxyl	288.8	1.6
	O1s O-C	532.6	23.8
	O1s O=C	531.0	2.1
**WS LCNF High E**	C1s C-C	284.6	31.8
	C1s C-O	286.2	27.0
	C1s O-C-O	287.8	8.0
	C1s carboxyl	289.0	1.3
	O1s O-C	532.6	27.3
	O1s O=C	530.9	1.5
**Flax LCNF low E**	C1s C-C	284.6	29.7
	C1s C-O	286.3	27.9
	C1s O-C-O	287.8	8.7
	C1s carboxyl	289.0	1.5
	O1s O-C	532.6	27.4
	O1s O=C	531.0	2.2
**Flax LCNF high E**	C1s C-C	284.6	36.9
	C1s C-O	286.3	25.1
	C1s O-C-O	287.7	7.8
	C1s carboxyl	288.8	2.0
	O1s O-C	532.6	25.9
	O1s O=C	530.7	1.0
**Hemp LCNF low E**	C1s C-C	284.6	37.1
	C1s C-O	286.2	23.8
	C1s O-C-O	287.7	8.0
	C1s carboxyl	289.0	2.0
	O1s O-C	532.6	24.2
	O1s O=C	531.0	2.2
**Hemp LCNF high E**	C1s C-C	284.6	21.6
	C1s C-O	286.2	30.8
	C1s O-C-O	287.6	10.0
	C1s carboxyl	288.9	2.5
	O1s O-C	532.6	33.3
	O1s O=C	530.8	0.6

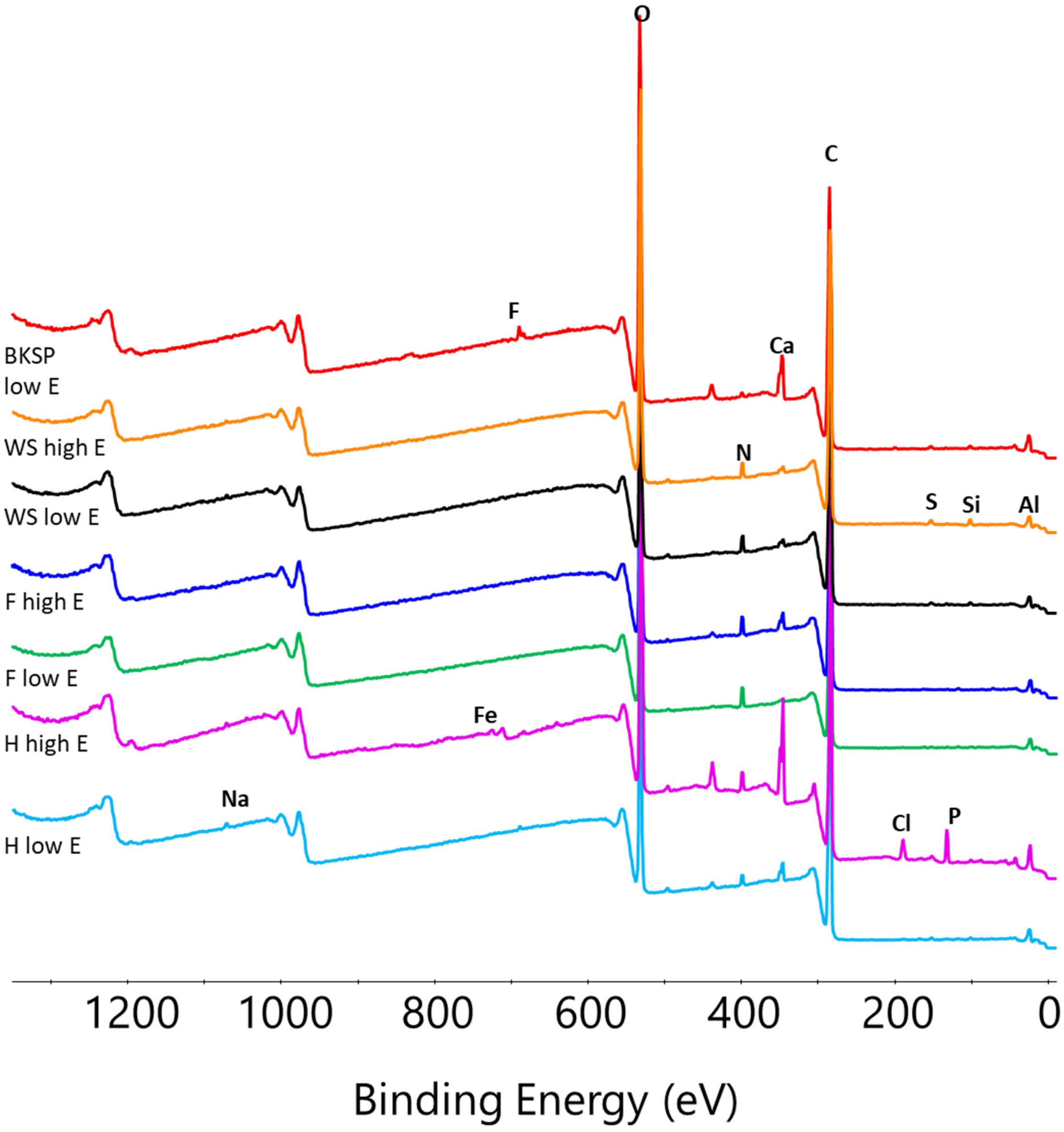

Figure S3 Entire XPS spectra of the various CNF and LCNF produced in this study. Note, only BKSP low energy is shown as both the low and high energy samples appeared the same on XPS.

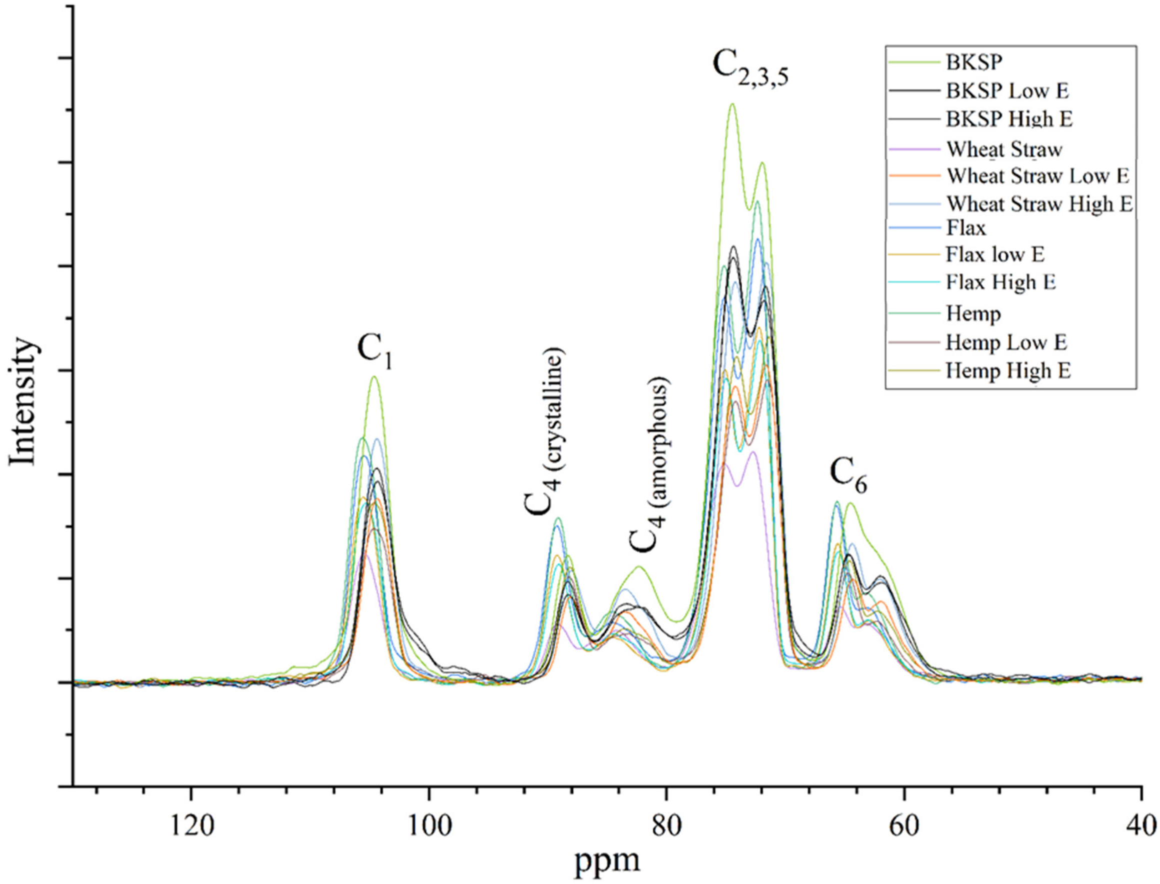

Figure S4. ^{13}C ss-NMR CP/MAS spectra of cellulose extracted from various biomass samples.

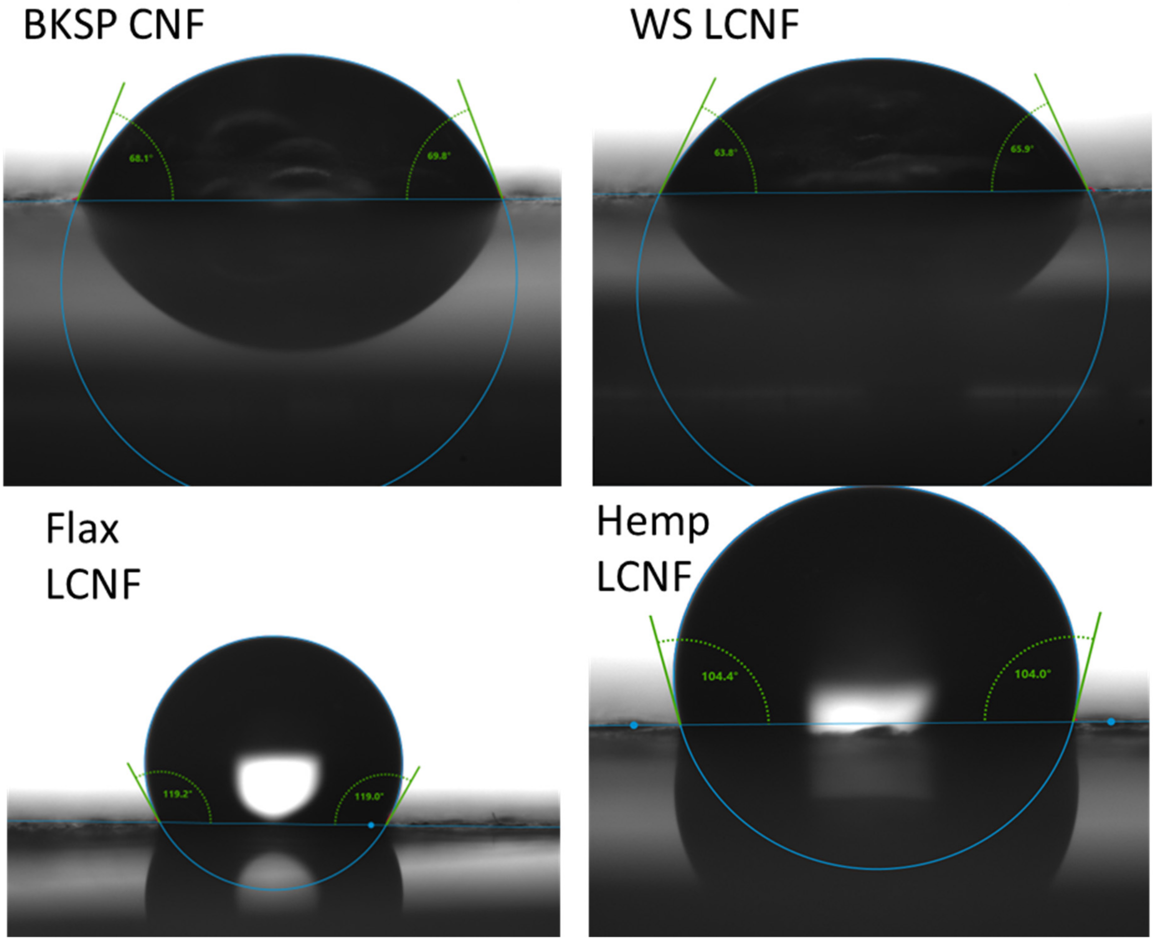

Figure S5. Water contact angle for the various CNF and LCNF produced in this work.
